# COVID-19 Resilience for Survival: Occurrence of Domestic Violence During Lockdown at a Rural American College of Surgeons Verified Level One Trauma Center

**DOI:** 10.7759/cureus.10059

**Published:** 2020-08-26

**Authors:** Heather X Rhodes, Kirklen Petersen, Laura Lunsford, Saptarshi Biswas

**Affiliations:** 1 Trauma, Grand Strand Medical Center, Myrtle Beach, USA

**Keywords:** assault, covid-19, domestic violence, coronavirus

## Abstract

Background

As the early peak phase in the coronavirus outbreak has intensified, stay-at-home mandates requiring identified individuals as nonessential were advised to remain home to prevent community transmission of the disease. Further mandates escalated isolated environments such as school closures, social distancing, travel restrictions, closure of public gathering spaces, and business closures. As citizens were forced to stay home during the pandemic, the crisis created intensifying stressors and isolation, which fostered an environment for increased domestic violence.

Methods

A retrospective review of all emergency department (ED) patients that presented to an American College of Surgeons (ACS) verified rural level one trauma center with associated diagnostic coding for assault was conducted during the *Coronavirus* 2019 (COVID-19) lockdown, integral dates March 16, 2020 to April 30, 2020. In particular the identification of proportional assaults that presented to the ED after school closures (March 16, 2020) was compared to the previous year (March 16, 2019 to April 30, 2019). The data collected included patient characteristics, grouping by mechanism, grouping by a specific mechanism, and domestic violence perpetrators.

Results

A statistically significant (p = 0.01) increase in assaults was found during the COVID-19 lockdown, particularly during the period after school closures.

Conclusions

Although overall trauma volume was reduced during the COVID-19 stay-at-home mandates, a significant increase in domestic violence assaults was observed. Largely the assaults were perpetrated against white men by partners and unspecified nonfamily members, which were predominantly penetrating injuries.

## Introduction

Global reports suggest a much higher incidence of domestic violence during the early peak phase of the *Coronavirus* 2019 (COVID-19) pandemic, as compared to previous years [1,2]. Some contributing factors known to increase the prevalence of domestic violence include social isolation, economic stressors, boredom, lack of control, fear, and lack of access to support measures [3,4]. The compounding factors related to quarantine conditions can be accompanied by psychological stressors such as depression, alcohol abuse, and post-traumatic stress symptoms [1]. A rapid increase in domestic violence has developed across the globe, with some countries reporting a doubling or tripling in rates of occurrence [1,2]. The lockdown scenario may increase the frequency or severity of existing domestic violence situations, particularly among first-time abusers. Rates of people committing domestic abuse for the first time during the lockdown are as high as 23% [5]. Additionally, several countries have reported massive increases in demand for help and support via phone lines, internet searches, and refugee websites [1,2,4]. Globally, the mental health effects of COVID-19 have resulted in profound increases in domestic violence and suicide; however, little is known regarding the impact of the pandemic among rural populations [6].

Despite well-documented increases in domestic violence cases during the pandemic overseas, few studies have looked at domestic violence trends in the United States, particularly in rural populations. Individual states report increases in domestic abuse incidents ranging from 21% to 35% [4]. Additionally, increases in domestic violence have referenced police reports and phone calls, rather than those presenting to health-care professionals, such as through the emergency department (ED) [2]. Common modes of injury and demographics of abusers have been overlooked, both of which are valuable in decreasing domestic violence during lockdown scenarios. The purpose of this study is to evaluate the incidence of domestic violence in a rural population during the early peak phase of the COVID-19 pandemic and compare total numbers of trauma and assault patients presenting to the ED, mechanisms of assault, and demographics of assault victims and perpetrators to the previous year.

## Materials and methods

This retrospective review of the early peak phase of COVID-19 during lockdown observed patients who presented to a rural American College of Surgeons (ACS) verified level one trauma center in South Carolina with an associated diagnostic code for assault, inclusive months March 16, 2020 to April 30, 2020. A comparison was made between assault patients during the COVID-19 lockdown and the previous year (March 16, 2019 to April 30, 2019). An international review board (IRB) exempt determination facilitated the data extraction from an integrated organizational repository. Assault definitions were based upon the Centers for Disease Control and Prevention (CDC) groupings by injury type [7]. Demographic characteristics included age, race, and gender. The patients were grouped by assault mechanism, such as assault by perpetrator, cut/pierce, firearm, struck by/against, other specified/not elsewhere classified (NEC), and unspecified. The groups by assault mechanism stratified into 18 specific groupings by a specific mechanism, such as assault by a knife. Lastly, domestic violence perpetrators were assessed and compared to the previous year. Assault patients in the early peak phase of COVID-19 during lockdown were compared to assault patients the previous year using a chi-square two-way test for homogeneity. This method used identified relationships between proportions of ED visits from the two populations of total trauma volume for the single assault categorical variable. 

## Results

**Figure 1:**
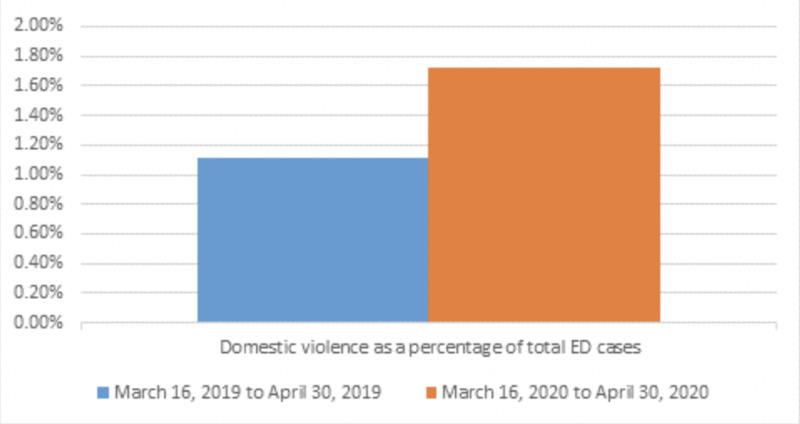
COVID Domestic Violence Trends Effects of domestic violence trends during COVID-19 lockdown (chi-square, p < 0.05).

A total of 2900 patients presented to the ED during the COVID-19 lockdown, and analysis of the dataset reported 50 patients with a cause code for assault. Similarly, the previous year captured 7008 ED admissions that included 78 patients identified as victims of assault. A statistically significant (p = 0.01) increase in assaults was found during the COVID-19 lockdown (Figure 1). Demographically this increase was associated with white (72%) males (62%; Table 1). The comparison years of 2019 to 2020 showed increased mechanisms of assault were largely penetrating, which included cut/pierce (2.6% vs. 18%) and knife (0% vs. 12%) injuries (Table 2). Blunt injuries from 2019 to 2020 were reduced (84.6% vs. 56%; Table 3). Domestic violence perpetrators by husbands during the COVID-19 lockdown showed a dramatic reduction during the study periods (33.3% vs. 0.0%; Table 4). Increases in domestic violence during the comparative years were largely associated with male partners (0% vs. 25%) and unspecified nonfamily members (0% vs. 25%). 

**Table 1:** Patient Characteristics IQR: Interquartile range.

Domestic Violence Totals by Year	2019	2020
Patient Characteristics	(N = 78)	(N = 50)
Age; Median [IQR] Mean ± SD	31.0 [19.2, 42.0]	31.0 [26.2, 42.0]
	33.1 ± 15.6	34.3 ± 12.4
Race: Black	28.2% (22)	22.0% (11)
Other	10.3% (8)	6.0% (3)
Unknown	1.3% (1)	0.0% (0)
White	60.3% (47)	72.0% (36)
Gender: Male	52.6% (41)	62.0% (31)

**Table 2:** Mode of Injury NEC: Not elsewhere classified.

Mode of Injury	2019	2020
Assault by perpetrator	3.8% (3)	8.0% (4)
Cut/Pierce	2.6% (2)	18.0% (9)
Firearm	2.6% (2)	2.0% (1)
Other specified, NEC	0.0% (0)	6.0% (3)
Struck by/against	84.6% (66)	56.0% (28)
Unspecified	6.4% (5)	10.0% (5)

**Table 3:** Grouping by Specific Mechanism

Mode of Injury Description	2019	2020
Assault by blunt object	10.3% (8)	4.0% (2)
Assault by handgun discharge	1.3% (1)	0.0% (0)
Assault by human bite	1.3% (1)	6.0% (3)
Assault by knife	0.0% (0)	12.0% (6)
Assault by other bodily force	5.1% (4)	0.0% (0)
Assault by other sharp object	1.3% (1)	2.0% (1)
Assault by other specified means	0.0% (0)	6.0% (3)
Assault by sharp glass	1.3% (1)	0.0% (0)
Assault by strike against or bumped into by another person	26.9% (21)	6.0% (3)
Assault by strike by baseball bat	1.3% (1)	0.0% (0)
Assault by unarmed brawl or fight	39.7% (31)	40.0% (20)
Assault by unspecified firearm discharge	1.3% (1)	2.0% (1)
Assault by unspecified means	6.4% (5)	10.0% (5)
Assault by unspecified sharp object	0.0% (0)	4.0% (2)
Husband, perpetrator of maltreatment and neglect	1.3% (1)	0.0% (0)
Male partner, perpetrator of maltreatment and neglect	0.0% (0)	2.0% (1)
Unspecified nonfamily member, perpetrator of maltreatment and neglect	0.0% (0)	2.0% (1)
Unspecified perpetrator of maltreatment and neglect	2.6% (2)	4.0% (2)

**Table 4:** Domestic Violence Perpetrators

Perpetrators	2019	2020
Husband, perpetrator of maltreatment and neglect	33.3% (1)	0.0% (0)
Male partner, perpetrator of maltreatment and neglect	0.0% (0)	25.0% (1)
Unspecified nonfamily member, perpetrator of maltreatment and neglect	0.0% (0)	25.0% (1)
Unspecified perpetrator of maltreatment and neglect	66.7% (2)	50.0% (2)

## Discussion

In the midst of crises such as COVID-19, domestic violence and similar issues are often neglected as attention is focused on fundamental resources such as ventilators [8]. Times of crisis are arduous for victims of domestic violence as they are facing additional stressors. Despite the increased need for support, there is often less support available as resources are directed away from shelters, helplines, and refugee websites [8]. Additionally, rates of domestic violence are likely higher than the reports suggest as not all victims have access to reporting devices such as computers and phones. During lockdowns, victims find it difficult to leave the house to seek help from friends, medical professionals, and other community members.

This study highlights the prevalence of domestic violence in a rural population and the importance of support resources, especially during lockdown situations like COVID-19. During this time, as many as three billion people in two hundred countries are sheltering in place; yet, little is known regarding the sociopsychology impacts in a rural community within the United States [3,7,8]. There is increased support for funding aimed at reducing domestic violence following the lockdown [9]. This increased awareness and media attention toward domestic violence may aid in implementing new protocols for addressing these issues in future lockdown scenarios [9].

There is a need to improve support programs that impact domestic violence victims for this pandemic and for associated future crises. Maintaining and prioritizing shelters, hotlines, websites, and other support services are critical during lockdown mandates [8]. It is crucial for the general public to understand the difference they can make simply by heightened awareness to the welfare of their neighbors and community members [8]. A number of rapid adaptations have occurred to combat domestic violence during the COVID-19 pandemic, which will likely shape the response of future supportive services to aid in the mitigation of similar outcomes in the future. 

Limitations

As consequence of the progression of patient chart closures, an extremely contracted time frame was available for data collection and processing. As a result, the study only captured 45 days of data during the early peak phase of the COVID-19 pandemic. It is envisioned that follow-up studies will use expanded time frames and larger patient numbers. Although this is a small study, the effect of the COVID-19 lockdown on domestic violence is significant in the rural community observed.

## Conclusions

Based on the study results, we conclude that COVID-19 has resulted in a significant increase in domestic violence. This includes mostly penetrating injuries directed at white males by partners and unspecified nonfamily members. Overall ED volume during the study time period was reduced, which can be expected with the lockdown in place as less people are exposed to common modes of injury. We anticipate this study can be used to determine the best methods to reduce domestic violence during lockdowns and ensure victims of domestic violence have access to the help and support they need.
